# Knowledge is power: understandings of accessibility from mental health service providers in ethnically diverse communities

**DOI:** 10.1080/28324765.2025.2512730

**Published:** 2025-05-28

**Authors:** Harriet Lawrence, Cathy Brennan, Cara Gates, Comfort Dangana

**Affiliations:** a School of Medicine, University of Leeds, Leeds, UK; b School of Health, Leeds Beckett University, Leeds, UK; c Blossom Foundation, Manchester, UK

**Keywords:** Ethnic diversity, service accessibility, racism in mental health care, qualitative

## Abstract

**Background:**

Ethnically diverse communities experience inequity across mainstream mental health services. Multiple explanations have been suggested as underpinning these, including: stigma, lack of cultural humility, inaccessible structures and widespread racism. The current study aimed to explore understandings of mental health service accessibility from the perspective of third sector service providers in the UK. Third sector organisations are those that are neither part of the public nor private sector, key examples include charities and social enterprises. In the UK, this sector provides the majority of community level mental health services. This enabled consideration of system-level barriers impacting service accessibility.

**Method:**

Semi-structured interviews were facilitated with 15 ethnically diverse participants, employed by 14 different third sector organisations. Interviews were analysed using reflexive thematic analysis.

**Results:**

Five themes were developed from the analysis: “knowledge is power”, “navigating the pathway to inclusivity”, “from cultural competence to cultural humility”, “deepening connection” and “building on a weak foundation”.

**Discussion:**

This study highlights the multifaceted understandings of service accessibility. Uniting perspectives was the necessity for services to proactively take responsibility for disseminating knowledge regarding service access to ethnically diverse communities, recognising that the availability of services is not equally learned. Participants highlighted the value of authentic connection, supported by a willingness from clinicians to self-reflect and challenge internal biases and assumptions. Mainstream services were encouraged to dismantle the institutionally racist foundations, challenge established power structures and meaningfully promote those with diverse cultural experiences to service leadership positions.

## Introduction

There are current inconsistencies regarding the most helpful and appropriate terms, phrases and acronyms used to describe ethnicity, with an absence of universal agreement, as language continues to evolve and be evaluated. In this study “ethnically diverse communities” is used to reference individuals who do not identify as White British, as it is empowering, inclusive and enables specific ethnicities to then be named when known.

Research widely indicates that individuals from ethnically diverse communities experience inequity within mental health services, contributing to the maintenance of discrimination, power imbalances and poor health outcomes (Arday, 2018; Chouhan & Nazroo, 2020). Globally, ethnically diverse individuals are more likely to receive diagnoses of severe, enduring mental health difficulties compared to White individuals (Asonye et al., 2020; Bignall et al., 2022; Grey et al., 2013; Shim, 2021). In the UK specifically, Black African individuals are over three times more likely, and South Asian individuals one and a half times more likely, to experience compulsory admission to hospital under the Mental Health Act (Bhui et al., 2015; Halvorsrud et al., 2018; Keating et al., 2002; Suresh & Bhui, 2006). These findings have remained consistent, with recent figures reporting Black individuals were almost five times more likely to be detained under the mental health act compared to White individuals (NHS Digital, 2021). In contrast, ethnically diverse communities are under- represented in services where support is sought voluntarily, with Black African and Black Caribbean individuals approximately half as likely to visit a general practitioner before an admission than White individuals (Halvorsrud et al., 2018; Myrie & Gannon, 2013). This inequity extends to interventions offered, with Black individuals less likely to receive psychological support and more likely to be prescribed psychotropic drugs than White individuals (Das-Munshi et al., 2018).

Several possible explanations account for these disparities, many of which highlight the inaccessibility of mental health services for ethnically diverse communities and the difficulties encountered when they are accessed. Broadly, services have been described as rigid, inflexible and lacking in accountability (Keating et al., 2002). In a qualitative study exploring barriers to accessing mental health services in England, Memon et al. (2016) reported that structural barriers, including long wait times, led to difficulties escalating before being responded to. Individuals who struggled to articulate their health problems using particular language, in some cases, received inappropriate referrals and interventions. This has since been replicated, adding that language and communication barriers were especially prevalent for participants for whom English was a second language, with fears this could lead to miscommunications or having to repeatedly explain difficulties (Arday, 2018).

A frequently overlooked explanation relating to disparities in access to mental healthcare is a lack of cultural humility within the practice of clinicians. Cultural humility refers to an individual’s commitment to ongoing self-reflection, recognising the impact of their implicit racial biases, alongside maintaining an openness to continually learn about different cultures (Lekas et al., 2020). It therefore recognises that learning does not have a fixed endpoint to be achieved as cultures continue to change and evolve. Mental health professionals have explained their reluctance to discuss ethnicity as underpinned by feeling anxious and uncomfortable, with fears of being labelled racist or offensive outweighing the perceived benefits of exploring cultural differences (Dogra et al., 2007; Mensah et al., 2021). This reluctance to move towards cultural humility can lead to racialised service users feeling burdened by having to teach clinicians about their lived experiences of racism, with concerns about whether they will be listened to and believed (Alang, 2019). Black and minority ethnic service users have also reported experiencing discomfort when sharing their experiences of racism, resulting in self-censoring to protect White healthcare professionals from becoming upset (Arday, 2022). The reported absence of cultural humility is a unifying concept that implies mental health services do not consistently enable belonging through curiosity and acceptance of difference, which is likely to impact the accessibility of services.

Racism, and its existence within services, has often been overlooked due to claims of objectivity, for example, within mental health diagnoses (Brown, 2003). However, slavery, colonisation and economic exploitations have all contributed to forming the foundations of mental health services (Kirmayer et al., 2021). Without recognition of the implicit racism underlying much current practice, ethnically diverse communities will continue to feel unsafe and less accepted in services (Schouler-Ocak et al., 2021). Without challenge to structures that have been developed to favour White populations, accessible services will never be a reality for ethnically diverse populations (Kolivoski et al., 2014). However, research relating to the impact of racism within mental health services has remained divided, with a recent government commissioned report concluding that it is not widely evidenced (Sewell et al., 2021). Alternatively, the report suggested the greater prevalence of mental health difficulties amongst ethnic minority groups, and less access to support, is due to individual environmental and socioeconomic factors. This fails to acknowledge how racist structures contribute to both mental health difficulties and socioeconomic disadvantage, whilst also shifting responsibility for inequity away from mental health services. A clear example of this is the enduring impact of the American Medical Association’s racist exclusion of Black physicians from their organisation, which, although rectified due to the Civil Rights Act of 1964, has resulted in fewer Black physicians working in the US (Shim, 2021). Furthermore, acts of racism and discrimination within medical research, understandably have led to significant mistrust of medical professionals and medical researchers amongst ethnically diverse communities (Shavers et al., 2002).

Largely, previous research has categorised ethnically diverse communities as a “hard to reach” group, reinforcing the narrative that the responsibility for accessing mental health services lies with individuals. This categorisation dismisses the impact of structural barriers preventing service accessibility, which are often present in NHS services. As a result, previous research has largely focused on the experiences of individuals, with insufficient exploration of service-related factors inhibiting accessibility. The current study therefore aims to understand accessibility of mental health services from the perspective of third sector services providing support for ethnically diverse communities in the UK. This is to recognise that services should take responsibility for ensuring accessibility rather than relying on individual level change. Although the NHS is the largest provider of healthcare in the UK, third sector organisations provide the majority of community mental health services, they are generally well regarded and people find the support easy to access (Newbigging et al., 2020). The current study, therefore, aimed to establish how ethnically diverse employees working within ethnically diverse third sector services understand accessibility and what they feel may impact accessibility for the communities they work with. Specifically, the research questions are:

- What do third sector service providers working with ethnically diverse communities believe an accessible service looks like?
- What can mental health services do to enhance accessibility for ethnically diverse communities?
- What are some of the barriers preventing service accessibility for ethnically diverse communities?

## Method

The study utilised a qualitative design using reflexive thematic analysis to analyse the data, attending to the nuances and complexities of accessibility of mental health services. The project received ethical approval from the School of Medicine Research Ethics Committee at the University of Leeds. The study was voluntary and written informed consent was obtained from all participants.

### Participants

Fifteen ethnically diverse participants took part in the study, aged between 25 and 66. Eleven participants identified as female, three identified as male and one identified as nonbinary. Participants worked in the third sector, including charities and community interest companies across England and Scotland, representing 14 organisations in total. All organisations provided mental health support to ethnically diverse communities and were also initially established by individuals belonging to that community. Some were more specific than others in relation to the communities worked with and interventions provided, for example, some services worked specifically with one ethnic group. Participants were given the opportunity to choose their own pseudonym or agreed to be assigned an appropriate name according to their heritage and cultural background. Demographic information relating to participants and their job roles are outlined in Table 1.

**Table 1.** Demographic information of participants

Participant Pseudonym	Self-description of Ethnicity	Self-description of Gender	Role in Service: Management/ Founder, Employee or Volunteer
Luna	Black African	Female	Employee
Amara	Black African	Female	Management/ Founder
Rani	British Asian Indian	Female	Management/ Founder
Tula	Black African	Female	Management/ Founder
Chidera	Black African	Male	Employee
Eshal	Kashmiri British	Female	Management/ Founder
Satvinder	Punjabi	Male	Management/ Founder
Yetunde	Black African	Female	Management/ Founder
Oni	Black African	Female	Employee
Lavindeep	Sikh	Female	Management/ Founder
Shahzad	British South Asian	Male	Management/ Founder
Hiranur	Turkish	Female	Employee
Khalil	Black African and White British	Nonbinary	Employee
Falaq	South Asian	Female	Employee
Zarina	Not disclosed	Female	Employee

### Recruitment procedure

In order to identify and recruit participants for the study, we utilised snowball sampling through previously established contacts in third sector mental health organisations. Further contact was made with additional services using email addresses that were publicly available online and were sourced using search engines and word of mouth from previous participants.

### Interview procedure

We chose to conduct semi-structured interviews as they support a conversational style, through using open-ended and variable questions, with further areas of exploration guided by responses from participants. Participants were given the option of attending the interview in person or virtually, using the video conferencing software, Zoom. We selected video conferencing as it allows for both audio and visual imaging in real time, whilst enabling access to a wider geographical reach of participants (Gray et al., 2020). All participants chose to attend the interview virtually.

We developed the interview questions collaboratively as a research team alongside an expert by experience, who was involved throughout the project. The interview topic guide had six main questions, including: “What do you believe an accessible service for ethnically diverse individuals looks like?” and “How does the service you work for facilitate accessible care for ethnically diverse individuals?” All interviews were audio recorded and transcribed to ensure the content of the conversations was captured accurately, to inform the subsequent analysis.

### Research team

Reflexive thematic analysis encourages the researcher to consider how their personal positioning, assumptions and values have informed and shaped the research project, as well as their relationship to the participants (Wilkinson, 1988; Willig, 2013). It is therefore important for me, as the lead researcher, and the wider research team, to outline our social context and identity transparently. I am a White British woman, working as a Clinical Psychologist in the NHS. Through my clinical work, I have become acutely aware of the under and over-representation of ethnically diverse individuals, depending on the type of service being provided. I have observed the impact of rigid, inflexible structures that disproportionately impact accessibility for ethnically diverse service users. Importantly, I recognise that I have not experienced racism or discrimination based on my ethnicity and through my employment I represent part of the NHS mental health system that my research is focused on. I wondered if this may hinder developing rapport and trust with participants within this study. However, my experiences did not reflect this and meeting with participants further instilled my responsibility to convey their views and share their stories in a powerful and meaningful way. I feel situating myself in a learner positioner and the participant in an expert position supported this process. This involved clarifying my understanding of responses and highlighting the expertise participants have of their services, which I hoped supported reducing any potential power imbalances within the interviews.

The wider research team consists of two White British women, who both work academically within university contexts, and a Black African woman who works for a charity providing mental health support for Black African women. As a research team, we discussed the evolution of language relating to ethnicity and the generalisations made across cultural groups and ethnicities. We also explored our individual identities, and I remained aware of how my own biases could impact the analysis process, particularly influencing what aspects of the data I was drawn to or may have overlooked. This will have been shaped by my ability to have overlooked many system-level barriers for much of my life, due to not being directly impacted by them. Therefore, the analysis process was facilitated collaboratively as a research team, recognising that within this team there were lived experiences of accessibility barriers within NHS services. My experiences will also have impacted how I wrote research documents, so their feedback was essential for mitigating against using culturally insensitive language or inappropriate terminology. We also self-reflected on our united positioning as a research team, in which we all recognise that institutional racism exists within services in the UK and want to learn more about how a systemic approach could support addressing oppressive structures. In addition, during the planning stages of the project, I met with an ethnically diverse network, working across UK-based services accessed by ethnically diverse communities. They provided valuable input including advocating for focusing on service-related barriers to accessibility and promoting an openness to understanding the breadth and depth of barriers to accessibility.

### Data analysis

The analysis was informed by a critical realist ontology, recognising an external reality in which racial oppression and discrimination of ethnically diverse communities exists across society. However, our understanding and interpretations of this reality are shaped by our sociocultural context and individual factors including, our experiences, language, culture and beliefs (O’Mahoney & Vincent, 2014). Reflexive thematic analysis acknowledges there is no direct relationship between reality and a person’s experience, through which meaning can be uncovered (Willig & Rogers, 2017). Alternatively, what is of interest is the person’s account of their experiences and their interpretations, followed by the researcher’s subsequent reflections and active role in generating themes and producing knowledge.

We followed the six-step method for analysing data developed by Braun and Clarke (2006). These stages were recursive, with repeated movement between them, alongside taking time away from the analysis and then revisiting it with renewed energy and creativity (Braun & Clarke, 2021). During the coding phase of analysis, I worked systematically through the data set noting meaningful quotes and ideas to form codes. A combined inductive and deductive approach was utilised, noting connections between the data and existing literature, whilst also understanding new ideas from the data. Coding at a latent level ensured the interpretations reached beyond the literal meaning of the data. Each code label was succinct and captured a single idea, for example, the quote “you feel like a part of something and included” was giving the code “forming community”. This process initially formed over 300 codes, although many of these were slight variations of the same concept. I, therefore, combined these to form a more workable and condensed number of coherent codes. The coding process was also facilitated alongside the wider research team, with the aim of discussing different interpretations and to share a richer, more in-depth awareness of the data, rather than seeking consensus. This involved each of us individually coding the same transcript, then meeting together to discuss our different interpretations. Connected codes then formed preliminary themes, capturing shared ideas and meaning. Within this stage, the code “knowledge is power” was promoted to a theme. There was also a clustering of codes relating to the idea of connection, both on a human level as highlighted in the code above “forming community” and at a systems level of the physical integration of services. These themes were further developed and refined, which supported clarifying their focus and ensuring they addressed the research questions. The themes were then named and reviewed, for example, initially we used the theme name “connection” yet realised this did not provide information about what about connection was important. What became clear was the importance of meaningful, in-depth connection, rather than just a surface level joining up of services, which led to the name “deepening connection”. When approaching the writing up of the themes, it felt important to ensure compelling data extracts were highlighted from across the entirety of the data set, representing all participants, whilst balancing the extracts with interpretative narrative (Braun & Clarke, 2022).

## Results

**Figure 1.**
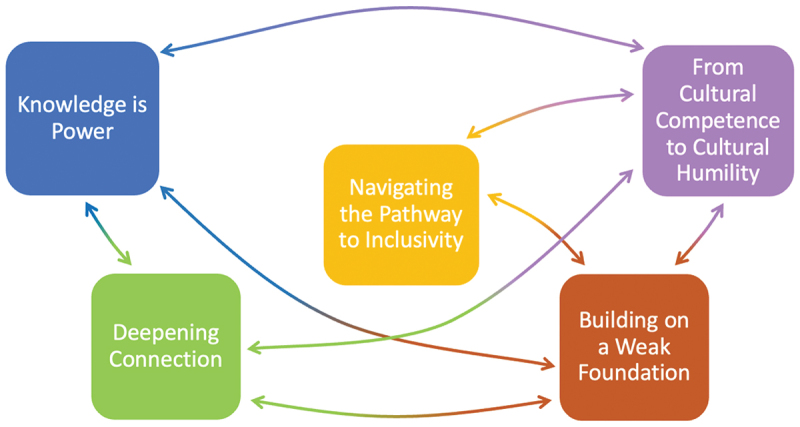
Thematic map.

Five themes were developed, named: knowledge is power’, “navigating the pathway to inclusivity”, “from cultural competence to cultural humility”, “deepening connection” and “building on a weak foundation”. A visual representation of how the themes are interrelated is shown in Figure 1, and Table 2 demonstrates the spread of data contributing to each theme across transcripts and participants.

**Table 2.** Breadth and depth of themes across transcripts

Theme	Quotes (N)	Participants (N)
Knowledge is power	193	15
Navigating the pathway to inclusivity	82	10
From cultural competence to cultural humility	265	15
Deepening connection	205	14
Building on a weak foundation	144	12

### Theme 1: knowledge is power

Participants highlighted that understanding the availability of mental health services is a prerequisite to making services accessible, emphasising the power in knowledge. However, this knowledge was positioned as unequally learned, as ethnically diverse individuals often experience less exposure to services compared to White individuals.

Information could be out there, but you don’t know where to find it … people don’t know what is available to them. (Amara)

Participants suggested that mainstream mental health services should take responsibility to highlight their support, and who can access it, using considered approaches, such as going to places of worship and community events. A proactive approach was recommended by participants to support tackling the imbalance of power between those who have access this knowledge and those who do not.

Quickly on the hard-to-reach perspective: really, really dislike that term. The hard to reach versus hardly reached, or seldom heard. And so, for me that shift in paradigm of phrasing is really important … we need to think about the barriers of access rather than just placing blame on communities themselves. (Satvinder)

The phrasing of “hard to reach” suggests there is an intention from individuals to not access services and acts as a way of mainstream services placing responsibility within communities for the underrepresentation of ethnically diverse individuals.

Another aspect of this theme is navigating the language used within services and knowing the language nuances which lead to appropriate onward mental health referrals and responses within services. Many participants referenced a necessity to use the “right” words to receive this, which they had not been exposed to or taught.

There’s almost like the buzz word, what do you say to the GP. What do you say to the gatekeeper to get in … to let you into the services that you need, and my people don’t know the right words. (Lavindeep)

Participants positioned language as a social construct, which evolves, but continues to enforce discrimination, as some mental health terms cannot be translated across languages or are not accepted in certain cultures, emphasising the power in language.

Try changing the language to increase access, instead of calling it therapy, call it a support session or community support group as this terminology is less intimidating. (Hiranur)

### Theme 2: navigating the pathway to inclusivity

Defining inclusivity within mental health services, how inclusivity links to accessibility, and the ways it can be operationalised, formed differing views amongst participants. Some participants suggest that for a service to be inclusive, there needs to be initial specificity, with staff members trained to work with specific ethnically diverse communities and who understand the unique barriers that may have impacted them.

I’d say there’s a pressure to be inclusive at the surface level. Because ultimately our service is very inclusive and reflective to folk with different experiences but all within [specific ethnicity] communities. But if you push that inclusivity at the first level, that can come at a detriment to the folks that you’re really trying to work with. (Satvinder)

Most participants identified that developing accessible services involves providing tailored and flexible support. However, it seems meeting the specific cultural needs of ethnically diverse service users then led to these services being perceived as welcoming and accessible to individuals with ethnic identities wider than the original focus of the service. This suggests that prioritising initial exclusivity results in movement towards inclusivity.

It was initially a charity to support [specific ethnic group] but like I said we now work with anyone who needs crisis and support. If someone is coming to ask for help, most of the time they really need it and it’s taken a lot for them to come here. (Luna)

Sometimes people like to work with a therapist who are the same culture you know or language as them, but sometimes they don’t want to see people from same culture or same background, and we give the people choice. (Hiranur)

### Theme 3: from cultural competence to cultural humility

Many participants outlined their observations of clinicians interpreting mental health difficulties within ethnically diverse communities through the lens of their pre-existing beliefs and assumptions. This has wide-reaching consequences, including misinterpreting cultural norms for symptoms of mental health difficulties, or dismissing mental health difficulties.

Because we talk with our hands we look like we’re being aggressive. And we’d get the strongest drugs because of this. (Oni)

Participants outlined how cultural stereotyping and viewing experiences from a “White” lens led to experiences of racism being overlooked by mental health clinicians. They highlighted a need for curiosity and openness when working with all people.

She got handover from a White colleague who said this lady here has been pulling out her hair; she’s been speaking gibberish and she refuses to eat … What the woman was doing was removing her braids and doing her braids over, she was fasting, and she was praying … nobody bothered to ask why. (Tula)

Participants referred to the importance of mental health professionals developing a broad understanding of differences between cultures in relation to mental health difficulties and how these may impact service accessibility. This included the necessity for education regarding the history of racism within NHS services.

Having open and frank conversations about what has happened historically in the NHS and why there’s mistrust, rather than brushing over it. People want to move forward, but you can’t move forward without looking back and there’s a reason for mistrust. (Chidera)

There was an identified distinction between learning about how culture may impact the expression of presenting difficulties, whilst also being guided by service users in a person-centred, individualised way. Many participants referred to third sector organisations navigating this effectively in practice.

Look at your prejudicial judgement cause we’re all brought up with certain ideas, attitudes and judgements really unless you work through these things. You may have assumptions of other people or other races and cultures that can keep discrimination going. (Hiranur)

Don’t make me fit your narrative cause you went on a course three weeks ago. (Rani)

Rani implies that using education and training to justify assumptions can lead to White staff members maintaining their power in services through claiming an expert position. Alternatively, participants recommended adopting a continuous learner position, recognising that learning about one culture does not equate to understanding diverse populations.

### Theme 4: deepening connection

The theme “deepening connection” depicts a core idea that prioritising forming and maintaining authentic connection underpins accessibility, though connection was expressed in several ways. Participants described the value of listening and validating difficulties, incorporating a natural process of being human together.

If everybody could just learn to be more compassionate from a human perspective, then you could find a way of healing each other. (Eshal)

Enabling relatability was used to illustrate how connections are deepened through unity and two-way sharing, moving away from the expected set up of “clinician” and “patient”.

I went to a stop smoking clinic about seven years ago. And the woman said to me, “Oh, yeah, the first few weeks are really hard”. and I was like, “Are they? When did you quit?” She goes, “I’ve never smoked!” I’m not coming back here again. How can you tell me the first two weeks are really hard when you’ve never even had a fag! (Rani)

Also central to forming connections is trust, and many participants implied that without it all other attempts of enhancing accessibility are meaningless. Developing trust incorporated clinicians recognising past experiences of racism within services and how mistreatment and deception of ethnically diverse communities has impacted their ability to be open and honest.

You can have a team of the best trained, trauma-informed, culturally aware, competent folks. But if you’re not getting people through the door because they’re unable to build that initial trust with you then it just won’t work. (Satvinder)

You go to your GP to say I’m going through abuse. And they ask you, “Oh, is it affecting your children?” and as a mother you say “yes, it’s affecting my children; my children cry”. Then what happens is social services. They can take your children. Now when a lady has experienced that how many do you think will go back to tell their GP same thing. No. So, that lack of trust is there. (Yetunde)

Enabling connection can also be more practically focused, incorporating joining up services and collaborating via two-way knowledge sharing. This involves acknowledging the strengths of different services and the skills within them.

What we realised is by working with community leaders and Imams, they’re the first point of access for a lot of people who are struggling. Cause there’s that level of trust there. (Falaq)

The ease of access to community organisations, due to preestablished relationships of trust, was referred to. However, participants acknowledged that many community-led organisations are not equipped with skills to explore mental health difficulties.

### Theme 5: building on a weak foundation

The theme “building on a weak foundation” encapsulates the service-related, systemic barriers to accessing services for ethnically diverse communities, which requires redesigning the foundations they are built on. Structural barriers identified were extensive, including poor funding, resource limitations, long wait times and navigating complex systems.

I’ll just figure it out myself because by the time you get seen, well if it was a broken leg, it would have to be gone and amputated. (Luna)

You could ring, you’re number 19 in the queue and who can hang on? (Zarina)

Participants expressed doubts regarding the motivations underpinning current attempts to increase accessibility, whereby mainstream services are doing little more than increasing visible representation of ethnically diverse employees in a tokenistic way. Increasing accessibility meaningfully was presented as embracing different cultures, using their ideas, and a willingness from services to give up power so representation can have a transformational impact.

It’s a mainstream service, catered to the mainstream, which is White British, cisgender, straight people and anyone outside of that is getting a different quality service because it’s not designed for them, and it’s not adapted for them either. (Khalil)

You follow the norms and culture wherever you work, even if you are from a diverse culture, if you work for the NHS you have to adopt their culture and structure as you can’t change that, the whole system would need to change. We are getting more user representation and getting better at understanding the needs of different cultures, but structurally they still remain the same, the issue of racism is still embedded within the service. (Hiranur)

Many participants suggested that the changes required to ensure mental health services are accessible are too extensive for small-scale adaptations and require complete reform.

Sometimes we have to look at our service and see how it’s built and that means some dismantling has to happen and I don’t think people feel comfortable dismantling things, it’s almost like let’s try and bolt something on. Ultimately, the system fundamentally doesn’t work. (Chidera)

An overarching idea across the data seemed to imply that the changes mainstream mental health services are willing to make do not align with current needs. Participants expressed this through outlining the intersecting nature of presenting difficulties experienced by individuals. Mental health services were described as addressing difficulties in isolation, rather than offering holistic, collaborative care, working alongside existing organisations. Participants outlined that if essential survival needs are not being met first, it is impossible for mental health interventions to be approached or succeed in supporting people helpfully.

It is never one simplistic problem, a person could be having issues with employment, their money, parenting, raising their children without any support networks. (Amara)

I need food. I need clothes. So, until that is met nobody can even talk about their emotion… if I’m poor and I’ve got my £5 I’m not spending it on travel to come … and if they can’t get there the NHS will think, oh, we offered her this and she hasn’t come. (Tula)

## Discussion

This research project aimed to explore understandings of mental health service accessibility for ethnically diverse communities from the perspective of third sector service providers working alongside these communities. The focus of the data collection aimed to address what participants believe an accessible service looks like, how services could become more accessible and identify some of the barriers preventing accessibility. Consistently, participants situated knowledge of mental health services as the first required stage of increasing service accessibility. However, through highlighting how this knowledge is unequally distributed, participants depicted knowledge as a tool to maintain power, as many ethnically diverse communities have limited exposure to service availability. A lack of knowledge of service availability within ethnically diverse communities has been widely documented both within the UK and internationally, making the pathway to mental health support more challenging (Memon et al., 2016; Myrie & Gannon, 2013; Rabiee & Smith, 2013; Yang et al., 2020). Addressing this power imbalance requires proactivity from mainstream services, through taking responsibility for disseminating awareness and partnering with ethnically diverse communities. This links to the theme “deepening connection”, whereby participants expressed that integrating services can address the unevidenced “hard to reach” label ascribed to ethnically diverse communities. Practical collaboration of services could incorporate sharing skills and equipping each other, recognising the dual responsibility for achieving the shared goal of providing accessible care. Enhanced co-production of services and including ethnically diverse communities at the centre of this process, has consistently improved engagement and intervention outcomes, as well as ensured services accommodate the values of ethnically diverse communities (Brach & Fraserirector, 2000; McEvoy et al., 2017).

Participants differed in their perceptions of how inclusivity within services should be attained, with some advocating for specialist services led by clinicians with a specific, shared cultural understanding and background. This aligns with findings suggesting that when racial discrimination has been experienced within healthcare settings, ethnically diverse individuals prefer to see professionals who identify as the same ethnicity, as they can relate to the impact of these experiences (Malat et al., 2010; Memon et al., 2016). A possible explanation of this suggests that relatability, in the form of shared ethnicity, acts as a protective factor against assumptions and harmful stereotypes portrayed about ethnically diverse communities (Moore et al., 2022). In this study, however, other participants suggested that a willingness to adapt services to meet different cultural needs creates a service ethos that is attractive to all. Uniting perspectives was the need for service flexibility to accommodate individual preferences and promote belonging, recognising that ethnically diverse communities are not homogenous. This closely links to the underpinnings of cultural humility which centralise individualised approaches, with clinicians engaging in ongoing exploration of diverse understandings of mental health difficulties, incorporating cultural needs within interventions. The theme “from cultural competence to cultural humility” acknowledges how making assumptions and interpreting difficulties through the lens of clinicians’ existing beliefs forms a barrier to service accessibility, a finding that has been widely supported (Rabiee & Smith, 2013). This theme also brings to light many systemic factors continuing to make mainstream services inaccessible, recognising that ethnically diverse communities are not homogenous in terms of their mental health support needs, and that cultural identities intersect with other factors such as age, gender, socio-economic status and religious belief, all of which may uniquely shape help-seeking behaviour (Bhui & Sashidharan, 2003).

The data suggests that these participants believe broad education and awareness of cultural norms are required to address stigma, prevent the enactment of cultural assumptions and develop trust with ethnically diverse communities. This links to the power of language outlined in the first theme, whereby participants recommended ensuring the terminology used to describe mental health interventions is accepted across cultures. McEvoy et al. (2017) illustrated comparable conclusions, reporting that using more acceptable terminology for mental health interventions within Jewish culture such as “self-improvement interventions” enhanced accessibility for this community. Respecting differences in cultural values, alongside considering the intended audience of interventions, should guide decisions regarding language used within services. These themes collectively introduce that power is not equally distributed within services and addressing this requires moving away from White British culture being the accepted norm and all other groups forming the contrast (Parekh, 2019).

A clear idea uniting the views of participants is the significance of belonging and the necessity for mental health services to promote acceptance and authentic connection. Belonging aligns with journeying towards cultural humility, through working collaboratively, whilst being led by service users, positioning them as the experts of their experiences. Participants indicated that to aid accessibility clinicians should adopt a continuous learning stance, self-reflecting on the impact of their internalised prejudice, whilst not using increased knowledge to reinforce their power. There was a clear distinction identified between cultural humility and cultural competence, which alternatively refers to developing skills and knowledge regarding cultural differences, and is content focused, situating knowledge as a goal to complete (Kirmayer, 2012; Shepherd, 2019).

Participants depicted cultural humility as attending to all aspects of a service user’s identity. This has been supported by previous research (Lazaridou & Heinz, 2022), in particular with literature related to counselling and psychotherapy showing the potential for cultural humility to strengthen therapeutic alliances (Coleman et al., 2024). Cultural humility also transcends across both individual and systemic level recommendations for change. Focusing on cultural humility involves considering the impact of racism within services, critical reflection from practitioners and empowering service users to share their stories, to reframe where power and responsibility for mental health difficulties lies (Bogle et al., 2021). A closely linked product of cultural humility is deepening connection, which forms another theme in the analysis. Participants centralised “being human”, which is achieved through authentic, genuine connection, built on compassion and trust. Building rapport and recognising the individualised preferences of service users, without being hindered by judgement, transcended across the analysis. Previous research studies have also illustrated the benefits of services prioritising interpersonal interaction, through developing trusting, confidential spaces, alongside engaging in open conversations about identity in a non-tokenistic way (Chew-Graham et al., 2002; Gurpinar-Morgan et al., 2014; Memon et al., 2016; Razai, Kankam, et al., 2021; Razai, Osama, et al., 2021). Furthermore, Acle et al. (2021) recommended collaborative, curious approaches to integrate culture within interventions, utilising culturally flexible psychological models and assessments to incorporate the client’s cultural worldview. This can ensure that there is greater safety within services, with an awareness of past experiences of racism and discrimination within services that have led to a lack of trust.

The final theme, “building on a weak foundation”, outlines the views that mainstream mental health services need rebuilding with anti-racist foundations. This contrasts to current, small-scale considerations, including shortening wait-times and forming clearer intervention pathways, which contribute minimally to increasing accessibility. The suggested foundations tie together most themes, including embracing cultural humility, increased service integration with greater involvement from local communities, promoting belonging and holistic provision of care. Participants referenced current tokenistic change, which lacks meaning and does little more than shift only visibility within the NHS. Patel and Keval (2018) powerfully depict the notion of visible representation without meaning as “window dressing”. This aligns with the suggestions of participants in the current study that mainstream services are doing “just enough” to communicate increases in diversity, without altering the foundation of services. Participants suggested that holistic, whole-system approaches need to form the heart of structural change, by promoting those with diverse cultural experiences into positions of leadership. Service accessibility reform needs to consider how institutional racism maintains socio-economic barriers to access, including the complex interaction of difficulties, such as poverty, leading to an inability to access services. Institutional racism impacts all themes in the analysis, prevents the enactment of person-centred care and is identifiable across systems in the UK. Participants suggest structural change to create services where all service users are valued and belong, providing a holistic, person-centred approach to care. These reforms must also be sensitive to the diversity *within* communities, avoiding one-size-fits-all solutions and instead ensuring that structural change is guided by consultation with a wide range of voices reflecting intersecting identities and lived experiences.

### Strengths

To our knowledge, this study is one of the first to explore understandings of mental health service accessibility from the perspectives of UK-based, third sector service providers, working with ethnically diverse communities. Through recruiting 15 participants representing 14 ethnically diverse services, the inaccuracy of the “hard to reach” terminology has also been addressed. This will hopefully promote ongoing engagement with third sector organisations within ethnically diverse communities, enacting the recommendation to increase service integration. Through shifting the focus of this study from service users to service providers, we were able to access system-level barriers and facilitators to accessibility which can support wider systemic change, and which may not have been identified through exploring the needs of a specific group.

### Limitations

Whilst the findings offer rich insights into participants’ perceptions of service accessibility, studying the perspectives of broad ethnically diverse participants meant we were unable to establish unique aspects of accessibility existing within specific communities. As a result, this study does not offer specific recommendations for services, nor outline a checklist of necessary requirements to achieve accessible services for particular communities. What it does do is offer a blueprint to guide system-led thinking in the development or redesign of services.

In this study, participants shared their reflections on factors they believe impact service accessibility for ethnically diverse populations. However, these may not equate to the experiences of those accessing their services and only reflect the view of third sector service providers. In addition, these findings are time-stamped, situated in the current context, which will continue developing as understanding and service development progresses.

### Future Directions for Research

Exploring the perspectives of third sector service providers has developed new insights into understanding mental health service accessibility. However, future research should establish whether the perspectives of service users align with those of staff, to build on these findings. In particular, focusing on service users who only attend third sector services, as opposed to mainstream services, could develop the evolving understanding of accessibility barriers. Due to the nature of not attending services, it may be challenging to represent the views of individuals who do not currently feel able to access mental health services, however their perspectives are valuable and necessary. Through this study actively disproving the efficacy of the term “hard to reach groups”, it may be possible to recruit those underrepresented in health research, through researchers engaging in proactive recruitment methods. In addition, it may be useful to facilitate research exploring specific ethnically diverse communities to enhance understanding of within-cultural variation in help seeking and perceptions of service accessibility.

## Conclusion

Mental health service accessibility for ethnically diverse communities is multifaceted, requiring close consideration of the overt and subtle expressions of power that prevent accessibility. Developing services where ethnically diverse communities are valued and belong, through cultivating person-centred, authentic connection, should be prioritised. Increased integration of services and dismantling of current services will support restructuring racist power hierarchies within services and enable critical examination of both individual and service level biases which restrict access for many ethnically diverse communities. This study makes a significant contribution to the literature regarding mental health service accessibility, identifying that change should be led by the continuous and proactive journey towards cultural humility and redistribution of power.

## Data Availability

The participants of this study did not give written consent for their data to be shared publicly, so due to the sensitive nature of the research supporting data is not available.
